# Prognostic analysis of nimotuzumab combined with concurrent chemoradiotherapy for locally advanced cervical cancer: a multicenter real-world study

**DOI:** 10.1038/s41598-025-98359-4

**Published:** 2025-05-07

**Authors:** Jiwei Li, Tao Wu, Manbo Cai, Changjun Xie, Sijuan Ding, Wen Zou, Jia Yao, Jingjing Wang

**Affiliations:** 1 Department of Oncology, The Second Xiangya Hospital of Central South University, Changsha, China; 2 Department of Oncology, Xiangya School of Medicine, Changde Hospital, Changde, China; 3 Department of Oncology, The First Affiliated Hospital, Hengyang Medical School, University of South China, Hengyang, China; 4 Department of Oncology, The Second Affiliated Hospital, Hengyang Medical School, University of South China, Hengyang, China; 5 Department of Oncology, The central hospital of Yongzhou, Yongzhou, China; 6 Department of General Surgery, The Second Xiangya Hospital of Central South University, Changsha, China

**Keywords:** Locally advanced cervical cancer, Concurrent chemoradiotherapy, Nimotuzumab, Prognosis, Safety, Targeted therapies, Gynaecological cancer

## Abstract

Nimotuzumab is a monoclonal antibody against EGFR. The therapeutic efficacy of nimotuzumab in cervical cancer treatment remains inconclusive, with current evidence insufficient to establish a definitive clinical benefit. Therefore, this study compares the efficacy and safety of nimotuzumab combined with concurrent chemoradiotherapy (CCRT) and CCRT in locally advanced cervical cancer (LACC). Information on patients with stage IIB-IVA cervical cancer who received CCRT combined with nimotuzumab or CCRT alone at five cancer centers from January 2021 to December 2022 were collected. Propensity score (PS) matching analysis was used to compare nimotuzumab group and no-nimotuzumab group. Clinical outcomes were analyzed. There were 195 patients enrolled. The 2-year overall survival (OS) and progression-free survival (PFS) rates were 92.5% and 89.5%, respectively. The objective response rate (ORR) was 90.8%. There were 60 patients in the nimotuzumab group and 135 patients in the no-nimotuzumab group. The nimotuzumab group had a better CR rate (51.7% vs. 26.7%, *P* = 0.001) and ORR (98.3% vs. 87.4%, *p* = 0.015) compared to the no-nimotuzumab group. After PS matching, there were 54 patients in each group. There were no significant differences in 2-year OS rate and PFS rate between the two groups before and after matching. However, the nimotuzumab group had also a better CR rate and ORR compared to the no-nimotuzumab group after matching. The incidence of grade 3–4 anemia was relatively higher in nimotuzumab group than in no-nimotuzumab group after matching, while no difference was observed in other adverse events between the two groups. The combination of nimotuzumab and CCRT for LACC improved CR rate and ORR, without increasing side effects, which might become a potential treatment strategy for LACC patients.

## Introduction

Cervical cancer is a life-threatening disease that is the fourth most commonly diagnosed neoplasm and the fourth leading cause of cancer death in females. In 2020, there were approximately 604,000 new cases of cervical cancer and 342,000 deaths globally, and its treatment remains a challenge^1^. Locally advanced cervical cancer (LACC) accounts for about 37% of cervical cancer, including FIGO stage IIB-IVA, and the standard treatment was platinum-based concurrent radiotherapy and chemotherapy (CCRT)^2^. At present, the 5-year overall survival (OS) of LACC patients are about 70–82.5% following completion of CCRT, however a proportion of patients experience recurrence or metastasis^3,4^. As a result, there is a pressing need to explore potentially available treatment approaches for LACC patients.

Targeted therapy is an effective treatment option that can improve survival and reduce the adverse effects of cancer^5^. Epidermal growth factor receptor (EGFR) belongs to the ErbB receptor tyrosine kinases family and can regulate gene expression, development, cell growth, and differentiation. There is evidence that EGFR is overexpressed in 70–90% of cervical cancer, and thus can be considered an effective target for patients with cervical cancer^6,7^. Nimotuzumab (also known as h-R3) is a humanized monoclonal antibody against EGFR, which has been proven to have essential anti-tumor functions both in vitro and in vivo by inhibiting proliferation, affecting angiogenesis, and promoting apoptosis^8,9^. Compared to other anti-EGFR treatments, nimotuzumab has advantages of low toxicity and low rash rates in a variety of tumors^10^, such as glioma, pancreatic cancer, non-small cell lung cancer, and head and neck squamous cell carcinoma^11^. Nimotuzumab has been approved to be used in combination with radiotherapy for the treatment of EGFR positive stage III/IV nasopharyngeal carcinoma and has the effect of sensitized radiotherapy^12^. Most cervical cancers are also induced by virus and express EGFR, just like nasopharyngeal carcinoma. Previous studies had shown that the combination of nimotuzumab and CCRT could improve the therapeutic efficacy of cervical cancer, however, these studies were either single center study, outdated, or had small sample sizes^13,14^. Despite its potential, nimotuzumab has not yet been assessed in well-powered randomized studies for cervical cancer. Here, we conducted a multicenter retrospective study comparing the efficacy and safety of CCRT combined with nimotuzumab and CCRT alone in the treatment of LACC patients.

## Methods

### Patients

This multicenter retrospective study analyzed consecutive patients with histologically confirmed cervical carcinoma treated between January 2021 and December 2022 at five tertiary medical centers: the Second Xiangya Hospital of Central South University, Changde Hospital, the First Affiliated Hospital of University of South China, the Second Affiliated Hospital of University of South China, and the central hospital of Yongzhou.

The following patients were included in the study: (1) Histopathologically verified squamous cell carcinoma, adenocarcinoma, or adenosquamous carcinoma of the cervix. (2) FIGO 2018 stage IIB-IVA disease treated with either concurrent chemoradiotherapy (CCRT) plus nimotuzumab or CCRT alone, (3) Minimum 12-month follow-up with complete oncological records.

The following patients were excluded from the study: (1) Comorbid malignancies, active infections, hematologic disorders, or major organ dysfunction, (2) Prior cervical cancer treatment (surgery/radiotherapy/systemic therapy), (3) Metastatic disease (M1) or pregnancy.

The last follow-up was conducted in January 2024. All methods were performed in accordance with the relevant guidelines and regulations, and informed consent was obtained from all subjects and/or their legal guardian(s). The study was conducted in accordance with the Declaration of Hensinki and approved by the appropriate ethics review board of the five participating hospitals. The experimental protocols were approved by the ethics review board of the Second Xiangya Hospital of Central South University, Changsha, China.

### Treatment

All patients underwent platinum-based CCRT. The experimental arm received weekly nimotuzumab (200 mg) concurrent with radiotherapy. Chemotherapy regimens were individualized based on renal function and performance status: (1) Tri-weekly: Paclitaxel (135–175 mg/m²) + cisplatin (50–70 mg/m²) or carboplatin (AUC 4–5). (2) Weekly: Cisplatin (30–40 mg/m²). Sequential: 6 cycles cisplatin followed by 2 cycles paclitaxel/cisplatin.

Intensity-modulated radiotherapy (IMRT) was delivered using Varian 23EX linear accelerators with 6-MV photons, planned via Eclipse Treatment Planning System (v11.0). Gross tumor volume of the lymph nodes (GTVnd) includes positive lymph nodes, which was confirmed using computed tomography (CT), magnetic resonance imaging (MRI), or positron-emission tomography (PET), and the clinical target volume (CTV) included entire uterus, parts of vagina, parametrium, GTVnd and the regional-nodal basin. The planning target volume (PTV) was systematically delineated through a 7–10 mm isotropic margin expansion encompassing both the gross tumor volume (GTV) and clinical target volume (CTV), ensuring adequate coverage of subclinical disease and accounting for potential geometric uncertainties. Cone-beam CT was performed weekly. The CTV dose was 45–50 Gy with 1.8–2 Gy administered daily, and the GTVnd dose was 60 Gy with 2.4 Gy administered daily. After 10–15 fractions of EBRT, brachytherapy will begin. Patients received 2D intracavitary brachytherapy (iridium 192) with a dose of 30 Gy/5fx to point A (defined as 2 cm lateral to the central canal of the uterus and 2 cm above the cervical opening) once a week beginning at the later time of EBRT. The entire RT course (including EBRT and brachytherapy) was completed within 8 weeks.

Nimotuzumab was administered 200 mg once a week for a total of 6 weeks, starting on the same day as CCRT. The imaging examination was carried out 2 months after the end of radiotherapy.

### Data collection and follow‑up

Baseline demographic and clinical characteristics encompassing age at diagnosis, FIGO stage (2018), histological subtype, maximal tumor diameter, chemotherapy regimen, and pretreatment imaging findings (contrast-enhanced CT, pelvic MRI, or 18 F-FDG PET/CT) were prospectively recorded in standardized case report forms. Systematic tumor response assessment was conducted 8 weeks post-radiotherapy using RECIST 1.1 criteria through triple-phase contrast-enhanced CT scans. Follow-up after the completion of CCRT includes every 3 months in the first 2 years, every 6 months in the next 3 years, and every year thereafter. At each follow-up visit, physical examination, imaging examination, hematology examination or cervical cytology examination are selected according to the patient’s condition.

### Statistical analysis

All analyses were performed using SPSS 26.0 (IBM Corp., Armonk, NY) with statistical significance set at α = 0.05 (two-tailed). Overall survival (OS) was defined as time interval from pathological diagnosis to all-cause mortality or censoring diagnosis until the date of death or final follow-up. Progression‐free survival (PFS) was defined as survival from the date of diagnosis until the date of (1) disease progression, (2) relapse, (3) mortality from any cause, or (4) final follow‐up. ORR refers to the proportion of patient achieving complete/partial response (CR/PR) according to RECIST 1.1. Safety was estimated by the National Cancer Institute Common Terminology Criteria for Adverse Events (NCI-CTCAE), version 3.0.

Patients were classified into the nimotuzumab group and no-nimotuzumab group. Treatment outcomes were compared between the two groups after 1:1 ratio propensity score (PS) matching with the nearest neighbor matching method using calipers of width equal to 0.01 of the SD of the logit of the PS, and adjusted for age, BMI, chemotherapy regimen, and chemotherapy cycles. Categorical variables were analyzed using χ² or Fisher’s exact tests as appropriate. Survival distributions were compared via Kaplan-Meier methodology with log-rank testing. Multivariable Cox proportional hazards models generated adjusted hazard ratios (HR) with 95% confidence intervals.And it was confirmed that all methods were performed in accordance with the relevant guidelines and regulations.

## Results

### Patient characteristics

During the period from January 2021 to December 2022, 224 patients were collected in this research. The median follow-up time was 20 months (12–36months). The median age of patients was 57 years (32–90 years). There were 106 patients received doublet agent concurrent chemotherapy, and 60 patients received single agent concurrent chemotherapy, and 29 patients received single + doublet agent concurrent chemotherapy. The median chemotherapy cycle is 4. All patients completed external irradiation and brachytherapy. Patients’ characteristics are summarized in Table 1.

**Table 1:** Clinical characteristics of patients.

Variable	Overall (%)	Before matching			After matching		
		Nimotuzumab (%)	No- Nimotuzumab (%)	*P* value	Nimotuzumab (%)	No- Nimotuzumab (%)	*P* value
Total	195 (100)	60 (30.8)	135 (69.2)		54 (50)	54 (50)	
Median BMI*	22.892	22.638	23.111	0.110	61	59	0.508
Median Age(y)	57	63	56	*0.021*	22.75	21.542	0.115
PS score*				0.957			0.696
0	10 (5.1)	3 (5.0)	7 (5.2)		3(5.6)	4(7.4)	
1	185(94.9)	57(95.0)	128 (94.8)		51(94.4)	50(92.6)	
Stage				0.093			0.521
IIB	77 (39.5)	22 (36.7)	55 (40.7)		22(40.7)	21(38.9)	
IIIA	3 (1.5)	1 (1.7)	2 (1.5)		1(1.9)	1(1.9)	
IIIB	36 (18.5)	6 (10.0)	30 (22.2)		5(9.3)	10(18.5)	
IIIC	78 (40.0)	30 (50.0)	48 (35.6)		26(48.1)	21(38.9)	
IIIC1	68						
IVa	1 (0.5)	1 (1.7)	0 (0.00)		1(1.9)	0(0.0)	
Tumor diameter				0.360			0.844
< 4 cm	72 (36.9)	25 (41.7)	47 (34.8)		21(38.9)	22(40.7)	
≥ 4 cm	123 (63.1)	35 (58.3)	88 (62.5)		33(61.1)	32(59.3)	
Pathology				0.651			0.153
Squamous carcinoma	190 (97.4)	58 (96.7)	132 (97.8)		52(96.3)	54(100)	
Adenocarcinoma	5 (2.6)	2 (3.3)	3 (2.2)		2(3.7)	0(0.0)	
Median cycle	4			*0.002*			0.700
< Median cycle	82 (42.1)	35 (58.3)	47 (34.8)		29(53.7)	27(50.0)	
≥Median cycle	113(57.9)	25 (41.7)	88 (65.2)		25(46.3)	27(50.0)	
Regimen				0.348			0.968
Doublet agent	106 (54.4)	28 (46.7)	78 (57.8)		28(51.9)	27(50.0)	
Single agent	60 (30.8)	22 (36.7)	38 (28.1)		10(18.5)	11(20.4)	
Single + doublet	29 (14.9)	10 (16.7)	19 (14.1)		16(29.6)	16(29.6)	

Significant values are in italics.

*BMI: Body Mass Index, ECOG: PS: Performance status.

The study cohort comprised 60 patients receiving nimotuzumab and 135 controls in the non-nimotuzumab group. Comparative analysis of baseline characteristics revealed some imbalances between groups, particularly in age distribution and chemotherapy cycles.

## Prognostic analysis and propensity score analysis

**Fig. 1:**
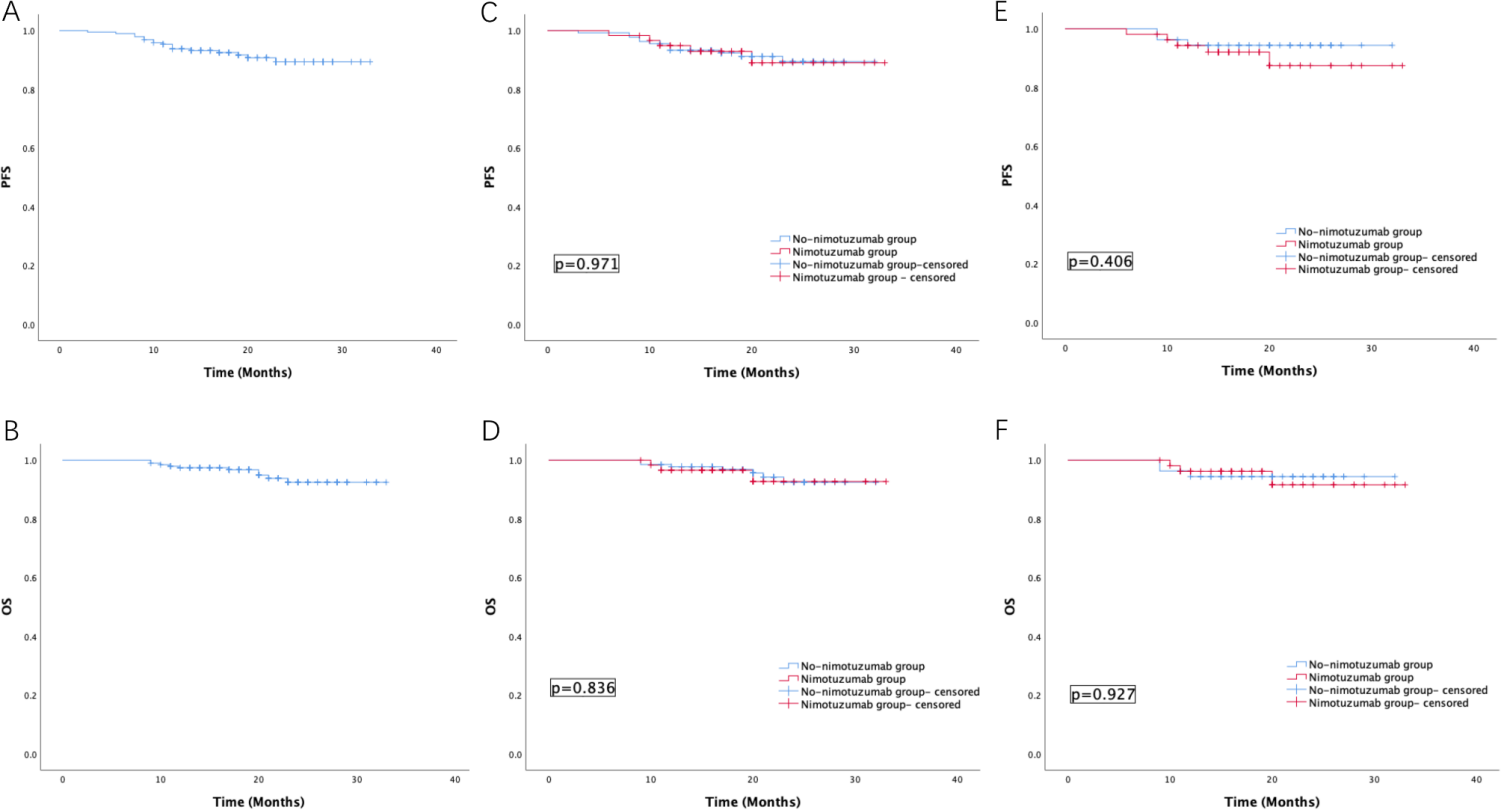
(**A**) Progression-free survival and (**B**) overall survival of all patients with LACC. (**C**) Progression-free survival and (**D**) overall survival of nimotuzumab group and no-nimotuzumab group before matching. (**E**) Progression-free survival and (**F**) overall survival of nimotuzumab group and no-nimotuzumab group after matching.

The 2-year OS and PFS rates of all patients were 92.5% and 89.5%, respectively (Fig. 1A, B). There was no statistically significant difference in PFS and OS between two groups (Nimotuzumab group vs. No- Nimotuzumab group: 2-year PFS: 89.1% vs. 89.4%, *p* = 0.971, OS: 92.7% vs. 92.5%, *p* = 0.836) (Fig. 1C, D). The CR rate and ORR of all patients were 34.4% and 90.8%, respectively. Patients who received nimotuzumab had better CR rate and ORR than those who did not received nimotuzumab (CR rate: 51.7% vs. 26.7%, *p* = 0.001, ORR: 98.3% vs. 87.4%, *p* = 0.015). Prognosis information was summarized in Table 2.

**Table 2:** Clinical treatment response of patients with or without nimotuzumab before and after matching.

Prognosis	Overall	Before matching			After matching		
		Nimotuzumab	No- Nimotuzumab	*P* value	Nimotuzumab	No- Nimotuzumab	*P* value
Treatment response				*0.001*			*0.003*
CR	67 (34.4%)	31 (51.7%)	36(26.7%)		28(551.9%)	12(22.2%)	
PR	110 (56.4%)	28 (46.7%)	82 (60.7%)		25(46.3%)	36(66.7%)	
SD	18 (9.2%)	1(1.7%)	17(12.6%)		1(1.9%)	6(11.1%)	
CR response				*0.001*			*0.001*
CR	67 (34.4%)	31 (51.7%)	36 (26.7%)		28(51.9%)	12(22.1%)	
Non-CR	128 (65.6%)	29 (48.3%)	99 (73.3%)		26(48.1%)	42(77.8%)	
Binary response				*0.015*			*0.003*
CR + PR	177 (90.8%)	59 (98.3%)	118 (87.4%)		53(98.1%)	48(88.9%)	
SD	18 (9.2%)	1 (1.7%)	17 (12.6%)		1(1.9%)	6(11.1%)	
2-year PFS rate	89.5%	89.1%	89.4%	0.971	87.5%	94.4%	0.406
2-year OS rate	92.5%	92.7%	92.5%	0.836	91.6%	94.4%	0.927

Significant values are in italics.

After PS matching, there were 54 patients in each group. All background factors between the two groups were well-balanced (Table 1). There was no significant difference between two groups after matching in terms of 2-year OS rate (91.6% vs. 94.4%, *p* = 0.927) and PFS rate (87.5% vs. 94.4%, *p* = 0.406) (Fig. 1E, F), however nimotuzumab group had a better CR rate (51.9% vs. 22.1, *p* = 0.001) and ORR (98.1% vs. 88.9, *p* = 0.003) than no-nimotuzumab group. At 2-year follow-up, the nimotuzumab-treated group achieved a local control rate of 95.0%, compared with 94.1% in the non-nimotuzumab group.

## Safety

In total, 176 patients included in the analysis of adverse events. There were 94.3% of patients experienced hematologic toxicity, of which 47.1% had grade 3–4 hematologic toxicity; 35.8% had nausea/vomiting, of which 1.7% had grade 3–4 nausea/vomiting; 36% had radiation proctitis, of which 1.7% had grade 3–4 radiation proctitis, 8.5% had radiation cystitis, and 32.4% had Fatigue.

After matching, only the incidence of grade 3–4 anemia was higher in nimotuzumab group than in no-nimotuzumab group, while there was no difference in the incidence of other adverse events between the two groups (Table 3). No drug-related severe AEs or deaths occurred.

**Table 3:** Adverse event profiles.

Adverse Effect	Overall (%)	Before matching			After matching		
		Nimotuzumab 59	No- Nimotuzumab 144	*P* value	Nimotuzumab 54	No- Nimotuzumab 54	*P* value
Hematologic	166(94.3)	50(90.9)	116(95.9)	0.744	45(91.8)	48(96.0)	0.439
G3-4	83(47.1)	26(47.3)	57(47.1)	0.984	26(53.1)	20(40)	0.193
Neutropenia	134(76.1)	36(65.5)	98(81.0)	0.106	31(63.3)	38(76.0)	0.281
G3-4	45(25.6)	12(21.8)	33(27.3)	0.442	12(24.4)	10(20)	0.591
Thrombocytopenia	79(45.1)	22(40.0)	57(47.5)	0.487	17(34.7)	22(44.0)	0.195
G3-4	15(8.5)	6(10.9)	9(7.4)	0.445	6(12.2)	3(6.0)	0.280
Anemia	139(79)	41(74.5)	98(81.0)	0.302	39(79.6)	39(78.0)	0.090
G3-4	43(24.4)	17(30.9)	26(21.5)	0.178	17(34.7)	8(16.0)	*0.032*
Nonhematologic							
Radiation proctitis	64(36)	26 (46.4)	38(31.1)	0.306	21 (42.0)	15(30.0)	0.363
G3-4	3(1.7)	1(1.8)	2(1.6)	0.944	1(2.0)	1(2.0)	1.000
Nausea/vomiting	63(35.8)	19 (34.5)	44 (35.4)	0.845	14 (28.6)	19 (38.0)	0.460
G3-4	3(1.7)	0(0)	3(2.5)	0.239	0(0)	2(4.0)	0.157
Radiation cystitis	15(8.5)	4(7.3)	11 (9.1)	0.773	4(8.2)	5 (12)	0.526
G3-4	0 (0)	0 (0)	0 (0)		0 (0)	0 (0)	
Fatigue	57 (32.4)	22 (40.0)	35 (28.9)	0.205	17 (34.7)	13 (26.0)	0.438
G3-4	2 (1.1)	0 (0)	2 (1.7)	0.338	0 (0)	1 (2.0)	0.320

Significant values are in italics.

## Discussion

EGFR can regulate cell proliferation and signal transduction, and can be implemented as a target for the treatment of various tumors^15^. As a humanized monoclonal antibody targeting EGFR, nimotuzumab has the effect of improving therapeutic efficacy in many tumors with acceptable toxicity, such as oesophageal cancer^16^, pancreatic cancer^17^, and head and neck cancer^18^. The synergistic mechanism of nimotuzumab is still unclear. The possible mechanisms are as follows: better improving the imbalance of T lymphocyte subsets and increasing the frequency of Tregs in peripheral blood of tumor patients^19^, blocking EGFR and inhibiting EGFR phosphorylation^20^, increasing apoptosis and G2/M phase arrest^21^.

In this study, it was found that nimotuzumab combined with CCRT improved the CR rate and ORR of LACC without increasing toxicity compared with CCRT alone. However, there were no significant difference between two groups in terms of 2-year OS and PFS rate. The follow-up time and sample were limited, which may be the reason why there is no difference in prognosis. CR and ORR reflected the short-term efficacy of treatment, and mainly evaluated the direct impact of treatment on tumors. They were very important indicators.

A prospective study of neoadjuvant chemoradiotherapy combined with nimotuzumab followed by surgery for LACC was conducted, and 36.4% had complete pathology response, the 2-year locoregional control rate, progression-free survival rate, distant metastasis-free survival rate, and overall survival rate were 95.0%, 85.2%, 84.0%, and 90.0%, respectively^22^. Another study compared the efficacy of nimotuzumab combined with CCRT with that of CCRT, and 120 patients were enrolled. The results showed the CR rate (86.15% vs. 70.91%, *P* = 0.040) and 3-year cumulative PFS rates (79.73% vs. 59.69%, *P* = 0.039) were significantly higher in the nimotuzumab group^13^. Another comparative study which enrolled 53 patients, showed that combining nimotuzumab with CCRT for the treatment of LACC resulted in extended median PFS (not reach vs. 27 months, *P* = 0.037) and higher CR rates (78.3% vs. 50%, *P* = 0.035) than CCRT alone^14^. The results of the previous studies were consistent with those of this study.

Another mouse and human chimeric anti-EGFR monoclonal antibody, cetuximab, has also been explored for the treatment of LACC. A randomized controlled Phase II study of 78 patients reported that there was no significant difference in DFS and OS between the cetuximab combined with CCRT group and CCRT group (2-year DFS: 63% vs. 76%, 2-year OS: 83% vs. 87%)^23^. Another single-arm study about cetuximab plus chemoradiotherapy for LACC revealed that 5-year PFS and OS rates for were 57.5% and 58.5%, respectively. The treatment was tolerable, and neoadjuvant cetuximab had an early response^24^. To date, no randomized controlled trials have been conducted to evaluate the efficacy of nimotuzumab in treating cervical neoplasms. Current evidence suggests that the therapeutic efficacy of cetuximab combined with concurrent chemoradiotherapy (CCRT) in locally advanced cervical cancer (LACC) remains limited, with a paucity of robust clinical studies. Large-scale randomized controlled trials are warranted to validate the clinical benefits of cetuximab in cervical cancer treatment.

Regarding the safety analysis, nimotuzumab showed a tolerable and acceptable toxicity profile. In our study, only the incidence of grade 3–4 anemia was higher in nimotuzumab group than in no-nimotuzumab group, while there was no difference in the incidence of other adverse events between the two groups after matching. The common AEs were hematological toxicity, nausea and vomiting, radiation proctitis, radiation cystitis, and fatigue, just like the toxicity of CCRT alone. Most AEs were generally manageable and no new toxicity signals emerged, with similar safety data in other studies^25^. A study on the treatment of LACC with cetuximab showed significant non hematological AEs, including grade 3 acneiform rash^24^. Cetuximab was reported with an acneiform rash of 80% in advanced cervical cancer in another study^26^. However, nimotuzumab lacks skin toxicity with a low rash rate, which is consistent with this study and other safety results^27^. In summary, our observation supports the favorable safety of nimotuzumab in the treatment of LACC population.

Nowadays, PD-1 inhibitors combined with CCRT are approved for the treatment of LACC. The phase III trial of pembrolizumab combined with CCRT in the treatment of LACC showed that the 2-year PFS of pembrolizumab + CCRT group were better than those of CCRT group (2-year PFS: 67.8% vs. 57.3%, *P* = 0.0020)^28^. However, the research of immunotherapy plus anti-EGFR antibody plus concurrent chemoradiotherapy in the treatment of LACC is still blank and needs further exploration.

There were some limitations in this study. First, this was a retrospective study, thus the bias cannot be ruled out. Second, the sample size was small, and the follow-up time was short. In addition, the variation of chemotherapy regimen in our study was another limitation. It is expected that a large sample size randomized controlled study will be carried out to verify the results of this study.

## Conclusion

The combination of nimotuzumab and CCRT for LACC improved CR rate and ORR, without increasing side effects. Consequently, the combination of CCRT with nimotuzumab holds potential as a reasonable therapeutic strategy for the patients with LACC and provides the basis for further exploration in LACC population.

## Data Availability

The authors agree to share anonymized data upon reasonable request by researchers. Someone wants to request the data from this study, please contact the corresponding author.

## References

[1] Sung, H. et al. Global Cancer Statistics 2020: GLOBOCAN Estimates of Incidence and Mortality Worldwide for 36 Cancers in 185 Countries. **71**(3):209–249. (2021). 10.3322/caac.21660 33538338

[2] Monk, B. J. et al. Proportions and incidence of locally advanced cervical cancer: a global systematic literature review. **32**(12):1531–1539. (2022). 10.1136/ijgc-2022-003801 PMC9763192 36241221

[3] Cohen, P. A., Jhingran, A., Oaknin, A. & Denny, L. Cervical cancer. *Lancet* **393** (10167), 169–182 (2019). 30638582 10.1016/S0140-6736(18)32470-X

[4] Zhang, Y. et al. Propensity score matching analysis to comparing cisplatin versus Nedaplatin based doublet agent concurrent chemoradiotherapy for locally advanced cervical cancer. *Sci. Rep.* **13** (1), 9352 (2023). 37291330 10.1038/s41598-023-36433-5 PMC10250460

[5] Zhou, Z. & Li, M. Targeted therapies for cancer. *BMC Med.* **20** (1), 90 (2022). 35272686 10.1186/s12916-022-02287-3 PMC8915534

[6] Schrevel, M. et al. Molecular mechanisms of epidermal growth factor receptor overexpression in patients with cervical cancer. *Mod. Pathol.* **24** (5), 720–728 (2011). 21252859 10.1038/modpathol.2010.239

[7] Soonthornthum, T. et al. Epidermal growth factor receptor as a biomarker for cervical cancer. *Ann. Oncol.* **22** (10), 2166–2178 (2011). 21325449 10.1093/annonc/mdq723

[8] Ramakrishnan, M. S. et al. Nimotuzumab, a promising therapeutic monoclonal for treatment of tumors of epithelial origin. *mAbs* **1** (1), 41–48 (2009). 20046573 10.4161/mabs.1.1.7509 PMC2715181

[9] Mazorra, Z. et al. Nimotuzumab: beyond the EGFR signaling cascade Inhibition. *Semin. Oncol.* **45** (1), 18–26 (2018). 30318080 10.1053/j.seminoncol.2018.04.008

[10] Garrido, G. et al. Sánchez-Ramírez B: upregulation of HLA class I expression on tumor cells by the Anti-EGFR antibody nimotuzumab. 8. (2017). 10.3389/fphar.2017.00595 PMC5635422 29056908

[11] Perez, R., Moreno, E., Garrido, G. & Crombet, T. EGFR-Targeting as a biological therapy: Understanding nimotuzumab’s clinical effects. **3**(2):2014–2031. (2011). 10.3390/cancers3022014 PMC3757402 24212794

[12] Yan Sun, C. H. et al. Jian-ji Pan, Jun Wang: Nimotuzumab plus chemoradiotherapy versus placebo plus chemoradiotherapy in patients with locally advanced nasopharyngeal carcinoma (NPC): A prospective, randomized-controlled, double-blinded, multicenter phase III clinical trial. *J Clin Oncol* 40(2022 (suppl 16; abstr 6001)). (2022).

[13] Zhang, L. et al. Efficacy and safety of nimotuzumab combined with chemoradiotherapy in the treatment of locally advanced cervical cancer. *Ann. Transl Med.* **10** (24), 1322 (2022). 36660627 10.21037/atm-22-5739 PMC9843379

[14] Chen, W. et al. Clinical study of nimotuzumab combined with concurrent radiochemotherapy for treatment of locally advanced cervical cancer. *Cancer Manag Res.* **11**, 8157–8165 (2019). 31564975 10.2147/CMAR.S191134 PMC6731987

[15] Shi, K. et al. Emerging strategies to overcome resistance to third-generation EGFR inhibitors. *J. Hematol. Oncol.* **15** (1), 94 (2022). 35840984 10.1186/s13045-022-01311-6 PMC9287895

[16] de Castro, G. Junior et al. A randomised phase II study of chemoradiotherapy with or without nimotuzumab in locally advanced oesophageal cancer: NICE trial. *Eur. J. Cancer*. **88**, 21–30 (2018). 29179134 10.1016/j.ejca.2017.10.005

[17] Qin, S. et al. Nimotuzumab plus gemcitabine for K-Ras Wild-Type locally advanced or metastatic pancreatic cancer. *J. Clin. Oncology: Official J. Am. Soc. Clin. Oncol.* **41** (33), 5163–5173 (2023). 10.1200/JCO.22.02630 PMC10666986 37647576

[18] Patil, V. M. et al. A randomized phase 3 trial comparing nimotuzumab plus cisplatin chemoradiotherapy versus cisplatin chemoradiotherapy alone in locally advanced head and neck cancer. **125**(18):3184–3197. (2019). 10.1002/cncr.32179 31150120

[19] Ao, M. et al. Changes in T lymphocyte subsets in peripheral blood of patients with middle-advanced cervical cancer before and after nimotuzumab combined with concurrent chemoradiotherapy. *J. Obstet. Gynaecol.* **43** (1), 2179915 (2023). 37001548 10.1080/01443615.2023.2179915

[20] Yu, Y. et al. Nimotuzumab, an EGFR–targeted antibody, promotes radiosensitivity of recurrent esophageal squamous cell carcinoma. *Int. J. Oncol.* **56** (4), 945–956 (2020). 32319582 10.3892/ijo.2020.4981

[21] Lin, S. et al. Sensitisation of human lung adenocarcinoma A549 cells to radiotherapy by nimotuzumab is associated with enhanced apoptosis and cell cycle arrest in the G2/M phase. *Cell. Biol. Int.* **39** (2), 146–151 (2015). 25044496 10.1002/cbin.10342

[22] Lu, H. et al. A prospective study on neoadjuvant chemoradiotherapy plus anti-EGFR monoclonal antibody followed by surgery for locally advanced cervical cancer. *OncoTargets Therapy*. **11**, 3785–3792 (2018). 29997439 10.2147/OTT.S164071 PMC6033113

[23] de la Rochefordiere, A. et al. PIK3CA pathway mutations predictive of poor response following standard radiochemotherapy ± cetuximab in cervical cancer patients. *Clin. cancer Research: Official J. Am. Association Cancer Res.* **21** (11), 2530–2537 (2015). 10.1158/1078-0432.CCR-14-2368 25724520

[24] Fracasso, P. M. et al. An exploratory study of neoadjuvant cetuximab followed by cetuximab and chemoradiotherapy in women with newly diagnosed locally advanced cervical cancer. *Am. J. Clin. Oncol.* **45** (7), 286–293 (2022). 35696702 10.1097/COC.0000000000000926 PMC9233135

[25] Yuan, Y. et al. Nimotuzumab combined with chemoradiotherapy for the treatment of cervical cancer: A meta-analysis of randomized controlled trials. 12. (2022). 10.3389/fonc.2022.994726 PMC9573994 36263226

[26] Hertlein, L. et al. Cetuximab monotherapy in advanced cervical cancer: a retrospective study with five patients. *Arch. Gynecol. Obstet.* **283** (1), 109–113 (2011). 20180130 10.1007/s00404-010-1389-1

[27] Rojo, F. et al. Pharmacodynamic trial of nimotuzumab in unresectable squamous cell carcinoma of the head and neck: A SENDO foundation study. *Clin. Cancer Res.* **16** (8), 2474–2482 (2010). 20371675 10.1158/1078-0432.CCR-09-3042

[28] Lorusso, D. et al. Pembrolizumab or placebo with chemoradiotherapy followed by pembrolizumab or placebo for newly diagnosed, high-risk, locally advanced cervical cancer (ENGOT-cx11/GOG-3047/KEYNOTE-A18): a randomised, double-blind, phase 3 clinical trial. *Lancet* **403** (10434), 1341–1350 (2024). 38521086 10.1016/S0140-6736(24)00317-9

